# Long-term visual outcomes and rehabilitation in Usher syndrome type II after retinal implant Argus II

**DOI:** 10.1186/s12886-018-0880-5

**Published:** 2018-08-22

**Authors:** Jeroni Nadal, María Iglesias

**Affiliations:** Barraquer Opthalmology Centre, International university of Cataluña, Barcelona, Spain

**Keywords:** Usher syndrome, Argus II, Retinitis pigmentosa, Rehabilitation

## Abstract

**Background:**

The aim of this article is to describe visual outcomes and posterior rehabilitation of the first Usher syndrome type II (USH2) patient receiving an Argus II (®) prosthesis.

**Case presentation:**

We present a case of a USH2 patient who underwent Argus II prosthesis surgery at the age of 53. He had hearing loss from birth and presented a very poor visual field with good light perception. He communicated through sign language translated by his interpreter, who explained all the information regarding the surgical procedure and who assisted in the posterior visual therapy.

Sixteen months after surgery, the patient communicates more fluently with sign language and is able to identify letters with high contrast over 6 cm and words up to four letters.

**Conclusions:**

This is the first case described in the literature of a USH2 patient receiving an Argus II prosthesis This is an alternative treatment for USH2 patients, whose interpreters are essential in the selection process and subsequent rehabilitation after surgery.

**Electronic supplementary material:**

The online version of this article (10.1186/s12886-018-0880-5) contains supplementary material, which is available to authorized users.

## Background

Usher syndrome type II (USH2) is a syndrome characterized by retinitis pigmentosa (RP) with significant visual reduction by the age of 50, sensor- neural hearing loss from birth and sometimes vestibular involvement [1]. It is clinically and genetically heterogeneous, and has an autosomal recessive inheritance [2].

The prognosis of USH2 patients is an unstable condition. They live isolated from their environment, practically from childhood, with a complete lack of hearing, speech and vision. Intelligible language development does not evolve in parallel with proper hearing, which necessitates the teaching from diagnosis of non-visual dependent rehabilitative strategies through sign language, involving hand contact with the help of an interpreter.

Sensory prostheses and cochlear implants help optimize speech but have minimal or insufficient effect if they are implanted after the age of 9 years [3]. However, the development of new visual technologies such as Argus (®) II Retinal Prosthesis System (Argus II) provides electrical stimulation on the retina enhancing visual perception in blind people. As previously described, this implant allows patients with RP not only to improve visual acuity, but also to increase their spatial perception and motor development [2]. Consequently, these implants open a new avenue of possibilities for patients cut off from the outside world such as those suffering from type ll Usher syndrome.

Post operative process of USH2 patients having an Argus II implant do not differ from other typical RP patients. The main difference is the challenge for these patients to complete rehabilitation after the surgery in the absence of auditory cue, but the interpreter has a fundamental role to minimize these difficulties. They serve as an interlocutor between patient and visual training technicians, in order to provide objective information about the physical environment, as well as being a mobility assistant.

We present the first USH2 case treated with an Argus II and his successful rehabilitation process with the help of an interpreter.

## Case presentation

A patient with sensorineural deafness and severe hearing loss from birth first came to our clinic at 8 years of age. He was diagnosed with RP, confirmed by electroretinography, and we suspected USH2. An interpreter or companion who knew sign language always accompanied him.

**Fig. 1:**
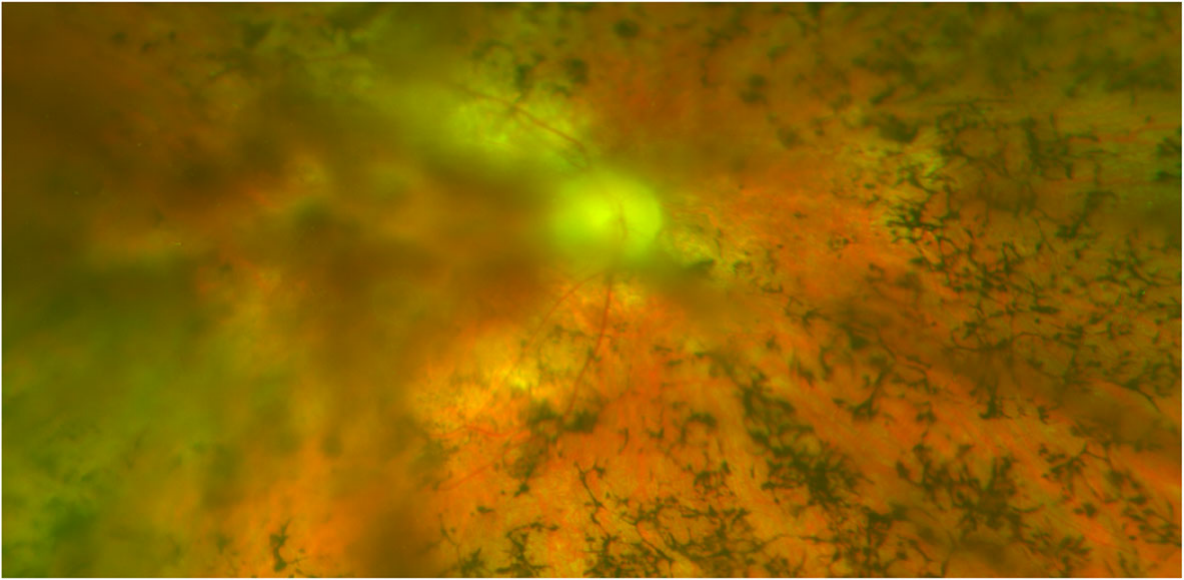
Funduscopy before surgery of the USH2 patient at the age of 53 with advanced retinitis pigmentosa

The RP evolved and in 2015, at the age of 53 years (Fig. 1), he had a visual field lower than 5° with good light perception. Consequently, we considered the patient a good candidate to receive an Argus II prosthesis.

The selection process and subsequent rehabilitation of patients eligible for this type of implant is a multidisciplinary task involving vision specialists and nonmedical support [3].

In this case, however, the process was made particularly difficult as we were unable to communicate with the patient. Communication was only possible through his interpreter, who always had to be situated in the center of his narrow field of vision.

As a result, all the information regarding pre- and post-op procedures had to be provided through the interpreter to the patient. He showed high capacity of comprehension and cooperation, therefore we were confident that he was able to fully understand all the conditions and characteristics of the pre-surgical preparation, the surgery and the requirements for post operative training. He was also aware of the importance of authorizing the publication of his case report.

**Fig. 2:**
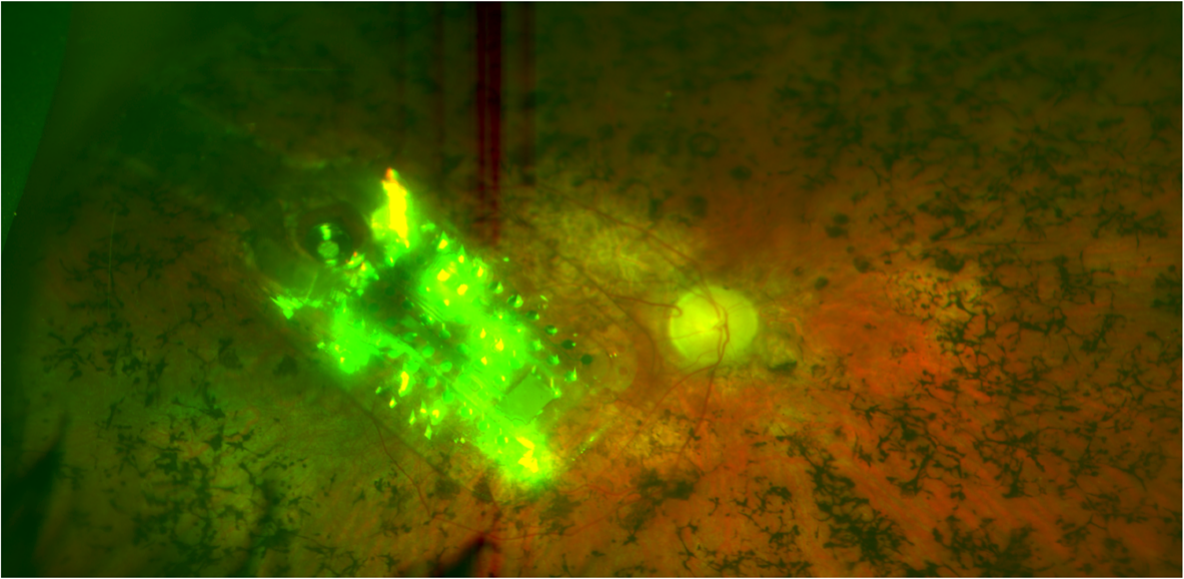
Funduscopy 1 month after surgery showing the Argus II implant in the posterior pole

**Fig. 3:**
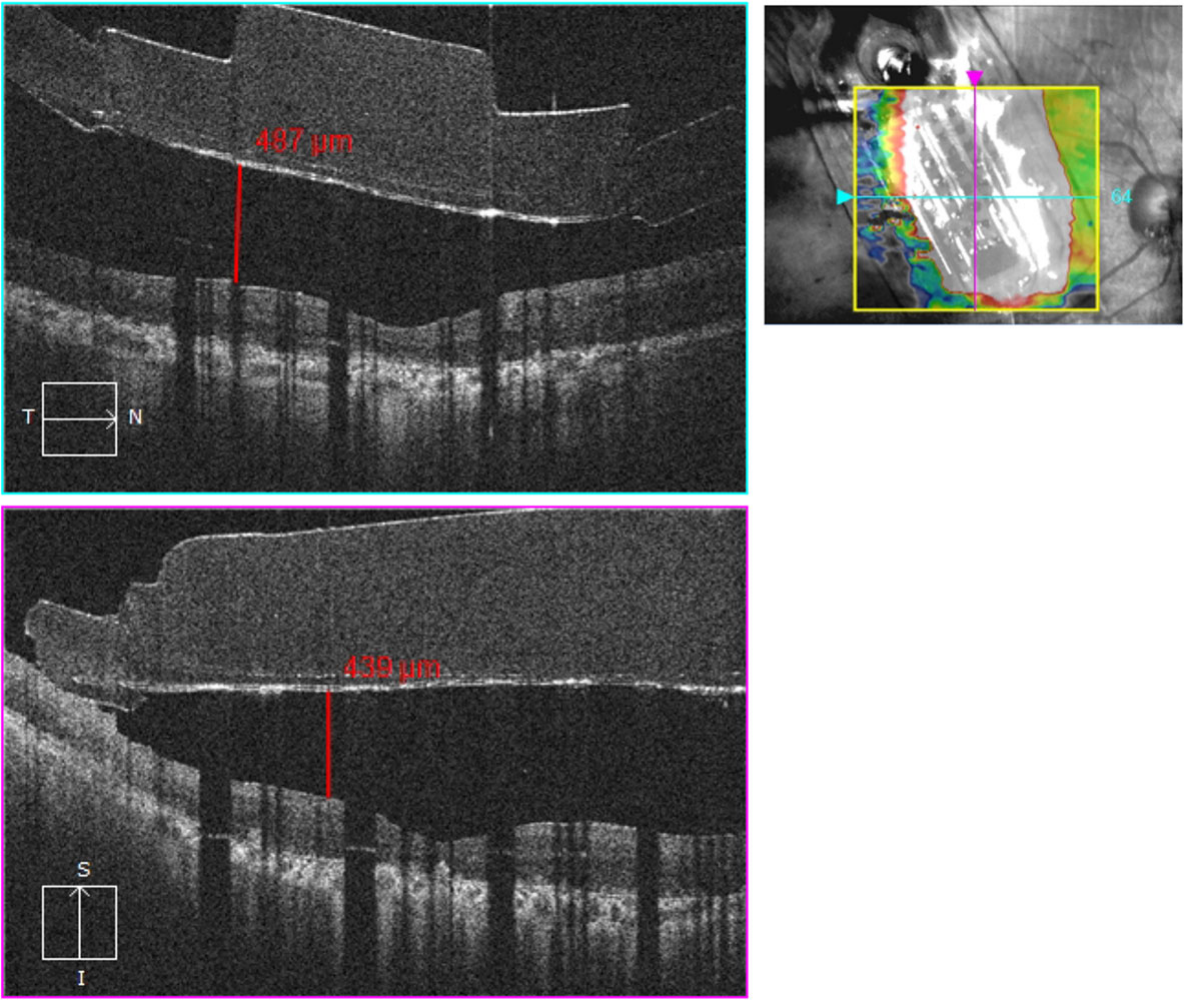
Optical Coherence Tomography (OCT) of the patient 1 month after the surgery. Macular OCT describes distance between the array and the macular inner retina. (Horizontal orientation- blue line, vertical orientation- pink line)

Surgery was performed as standard without any complications (Figs. 2 and 3), and the rehabilitation procedure was the same as that carried out with any other patient receiving an Argus II. The main difference was that an interpreter had to play a crucial role in the rehabilitation.

**Fig. 4:**
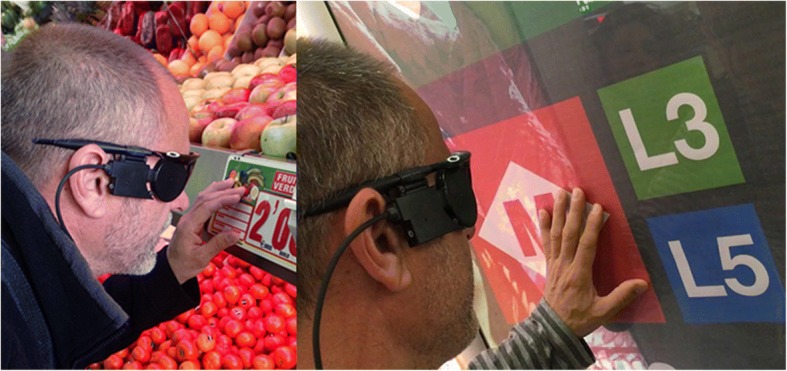
The USH2 patient in motility and orientation phase recognizing high contrast signs

To date, 16 months after surgery, our patient has a visual field angle of 15° and he is able to read letters with high contrast over 6 cm and words up to four letters at a 30 cms distance (Fig. 4).

The rehabilitation process began the third week after surgery, and consisted of two phases: a basic phase and an orientation and mobility phase.

In the basic phase the patient is trained in different sessions using an instructional kit provided by Second Sight training technicians. These sessions aim to teach patients to interpret and use new visual information to develop skills in their daily lives.

In this phase, our patient was taught how to locate, identify and recognize objects (such as detect lights, locate or identify objects, find utensils on a table, separate white and colored clothes, etc.) at three levels of difficulty. He was in a controlled environment with elements of high contrast and had to adapt to using head movements to obtain vision and sensitize a grey scale.

The objective of the orientation and mobility phase was for the patient to encounter real life situations using the system: locate parked or moving cars, bus stops and flagpoles, find doors, windows and elevators, follow a path or a sidewalk, crossing a pedestrian, etc.

This phase was carried out in the patient’s normal home environment and surroundings using his usual routes of movement (Figs. 1 and 2). The external environment (weather and elements) was assessed by the therapist who complemented the information signals and taught the patient how to use the Argus II implant filters according to varying environmental conditions.

## Discussion and conclusions

Sign language in USH2 necessarily entails the realization of a series of adaptations that modify this form of communication so that it is easier to perceive by the deaf blind person through its visual channel: adequate illumination, distance and position to see the hands of the interpreter.

When visual channel and visual acuity suffer a greater deterioration (as in our patient case), the patient is not able to continue perceiving the sign language in the same conditions, and they will need to constantly touch the hands of the interpreter.

An Argus II intervention in our patient has not only implied an improvement in vision but also in communication. The use of sign language has brought about an extraordinarily rapid adaptation and the patient performs daily activities without the need to use excessive hand contact. He can now distinguish the hand movements of his interpreter with increasing fluency whenever they are within his visual field (Additional file 1: Video S1). A similar capability was also demonstrated by Barry and Gislin [4] in their study of the Argus II retinal prosthesis for guiding fine hand movement with and without auditory feedback in 21 patients with RP.


**Additional file 1: Video S1.** The patient and his interpreter talking 16 months after surgery. (MP4 49378 kb)


One of the restrictions of the Argus II is the need for patients to learn how to point their head towards the object they want to see, and so, the optimal camera alignment position (CAP) may vary over time. Barry and Gislin [5] studied adaptation in three cases with intentional CAP misalignments, with and without auditory feedback for 5–6 months. Two of the subjects adapted to misaligned CAPs with auditory feedback while the other patient did not. They concluded that auditory feedback was necessary for successful adaptation to misaligned CAPs and regular recalibration of CAPs may be required to maintain hand-camera coordination.

In our patient, no camera alignment procedures were performed over a period of 16 months, and there is currently no evidence to suggest that further alignments need to be considered. However, given the fact that almost 200 patients have undergone Argus II implant procedures, we agree with Barry’s conclusion that further research in CAPs is necessary to determine optimal hand camera coordination.

It is important to highlight the crucial role played by the interpreter in explaining both the pre and postoperative procedures for this type of patient.

Today, our patient can perform tasks without touching objects, is able to communicate more fluently with sign language and he can identify words of up to four letters.

All this represents a dramatic change for a person who was isolated from the world for more than 20 years, and we can conclude that this is an effective, alternative pathway for USH2 treatment.

## Abbreviations

CAP: Camera alignment position
RP: Retinitis pigmentosa
USH2: Usher syndrome type II
